# The Effectiveness of Sodium-Glucose Cotransporter-2 (SGLT2) Inhibitors on Cardiovascular Outcomes and All-Cause Mortality in Patients With Acute Coronary Syndrome: A Systematic Review and Meta-Analysis

**DOI:** 10.7759/cureus.58019

**Published:** 2024-04-11

**Authors:** Tanya Sinha, Faria Khilji, FNU Laraib, Farhana Fatima, Mandeep Kaur, Sandipkumar S Chaudhari, Divine Besong Arrey Agbor, Areeba Khan

**Affiliations:** 1 Medical Education, Tribhuvan University, Kirtipur, NPL; 2 Internal Medicine, Tehsil Headquarter Hospital, Shakargarh, PAK; 3 Internal Medicine, Quaid-e-Azam Medical College, Bahawalpur, PAK; 4 Internal Medicine, Peoples University of Medical and Health Sciences, Nawabshah, PAK; 5 Internal Medicine, Dr. Rajendra Prasad Government Medical College, Kangra, IND; 6 Internal Medicine, Hospital Corporation of America (HCA) Florida Capital Hospital, Tallahassee, USA; 7 Cardiothoracic Surgery, University of Alabama at Birmingham, Birmingham, USA; 8 Family Medicine, University of North Dakota School of Medicine and Health Sciences, Fargo, USA; 9 Clinical Research and Internal Medicine, California Institute of Behavioral Neurosciences and Psychology, Fairfield, USA; 10 Internal Medicine, Richmond University Medical Center, Staten Island, USA; 11 Critical Care Medicine, United Medical and Dental College, Karachi, PAK

**Keywords:** sglt2 inhibitor, systematic review and meta-analysis, all-cause mortality, cardiovascular outcomes, acute coronary syndrome

## Abstract

The aim of this systematic review and meta-analysis was to investigate the impact of early sodium-glucose cotransporter-2 (SGLT2) initiation on long-term cardiovascular outcomes and all-cause mortality among patients with acute coronary syndrome (ACS). For this study, we adhered to the Preferred Reporting Items for Systematic Reviews and Meta-Analyses (PRISMA) 2020 guideline. Two researchers independently performed a comprehensive literature search on PubMed, Embase, and the Cochrane Library, spanning from the inception of each database to February 24, 2023, without language limitations. The outcomes examined in this meta-analysis comprised major adverse cardiovascular events (MACE) (as defined by individual studies), all-cause mortality, cardiovascular mortality, stroke (ischemic and hemorrhagic), recurrent ACS, and hospitalization due to heart failure (HF). A total of nine studies were included in this meta-analysis. The pooled analysis of nine studies revealed a significant reduction in the risk of MACE, all-cause mortality, cardiovascular mortality, and cardiovascular-related hospitalizations among patients receiving SGLT2 inhibitors (SGLT2i) compared to those in the control group. Additionally, there was a trend toward a lower risk of recurrent ACS in the SGLT2i group, although this difference did not reach statistical significance. The findings of this study suggest a promising therapeutic effect of SGLT2 inhibitors in this population. Further research, particularly focusing on myocardial infarction (MI) patients, is warranted to validate these results and potentially revolutionize ACS management.

## Introduction and background

Despite the progress made in early reperfusion therapy and medical interventions, acute coronary syndrome (ACS) continues to exert a substantial impact on global mortality and disability [1]. Epidemiological evidence indicates that over 40% of ACS patients suffer from diabetes, a condition independently associated with long-term major adverse cardiovascular events (MACE) among individuals at heightened cardiovascular risk [2]. ACS patients with diabetes frequently exhibit extensive coronary plaque buildup, larger lipid cores within these plaques, heightened macrophage infiltration, and increased plaque calcification levels [3], all contributing to an elevated risk of cardiovascular mortality [4].

Sodium-glucose cotransporter-2 (SGLT2) inhibitors have demonstrated efficacy in improving cardiorenal outcomes in patients with type 2 diabetes mellitus (T2DM), chronic kidney disease (CKD), and chronic heart failure with reduced ejection fraction (HFrEF). In the Empagliflozin Cardiovascular Outcome Event Trial in Type 2 Diabetes Mellitus Patients-Removing Excess Glucose (EMPA-REG OUTCOME) trial, empagliflozin exhibited favorable effects on cardiovascular mortality and reduced heart failure (HF) hospitalizations among T2DM patients with a prior history of myocardial infarction (MI) [5]. Given the expanding body of evidence across various disease conditions and the proposed mechanisms of action, it seems reasonable to explore the potential benefits of SGLT2 inhibition in improving outcomes for patients with ACS, particularly when initiated promptly after presentation [6]. The notion of early initiation and sustained use of SGLT2 inhibitors (SGLT2i) in ACS is alluring, given the multiple suggested mechanistic effects that could potentially alter the disease's natural progression and mitigate the risk of progressing to end-stage heart disease and chronic heart failure [7]. Recent investigations in both diabetic and nondiabetic experimental models of acute myocardial infarction have indicated the advantageous effects of SGLT2 inhibitors [8]. These potential mechanisms do not directly target coronary thrombosis but rather focus on mitigating reperfusion injury, reducing cardiomyocyte necrosis, and dampening neurohormonal activation [9].

SGLT2i have showcased their ability to decrease blood glucose levels by inhibiting glucose reabsorption in the proximal convoluted tubules of the kidney [10]. Numerous multicenter randomized controlled trials (RCTs) have established the cardiovascular advantages of SGLT2i among patients with T2DM at elevated cardiovascular risk. Nevertheless, these trials did not encompass patients in the initial stages of acute coronary events [11]. Given the potential benefits, it is theorized that patients with ACS could potentially gain advantages from the prompt initiation of SGLT2i therapy. To delve deeper into this hypothesis, we conducted a systematic review and meta-analysis aiming to investigate the impact of early SGLT2i initiation on long-term cardiovascular outcomes and all-cause mortality among patients with ACS.

## Review

Materials and methods

Literature Search

For this study, we adhered to the Preferred Reporting Items for Systematic Reviews and Meta-Analyses (PRISMA) 2020 guideline. Two researchers independently performed a comprehensive literature search on PubMed, Embase, and the Cochrane Library, spanning from the inception of each database to February 24, 2023, without publication date limitations. The search utilized keywords such as "SGLT2I" and "acute coronary syndrome" in addition to their synonyms and Medical Subject Heading (MeSH) terms. The search strategy for PubMed is found in the Appendices. Furthermore, we scanned the reference lists of selected studies to identify any additional relevant records.

Eligibility Criteria

This study encompassed randomized controlled trials and prospective or retrospective observational studies that fulfilled the following criteria: (a) the participants were aged 18 or above and diagnosed with ACS; (b) the experimental group was administered SGLT2 inhibitors, while the control group received either a placebo or another medication; and (c) we excluded studies involving patients other than those with ACS and studies published in languages other than English. Additionally, case reports, case series, and review articles were excluded. Furthermore, studies that did not report the necessary outcomes were also excluded.

Screening of Studies and Data Extraction

Two independent reviewers evaluated the titles and abstracts of articles, applying explicit inclusion and exclusion criteria. Subsequently, the full text of potentially relevant articles was obtained for a comprehensive assessment. Any disparities in determining study eligibility were resolved by a third reviewer. Data extraction was conducted independently by two reviewers, with a third reviewer validating the process. The collected data encompassed authors, publication year, country, study objective, design, sample size, follow-up duration, and outcomes. The outcomes examined in this meta-analysis comprised major adverse cardiovascular events (as defined by individual studies), all-cause mortality, cardiovascular mortality, stroke (ischemic and hemorrhagic), and recurrent ACS.

Statistical Analysis

For the data analysis, we utilized RevMan Version 5.4.1 (The Cochrane Collaboration, London, United Kingdom). To examine the impact of SGLT2 inhibitors on categorical outcomes, we calculated the risk ratio (RR) using random-effect models, employing Cochran-Mantel-Haenszel statistics along with their corresponding 95% confidence intervals (CI). Heterogeneity among the studies was evaluated using the I^2^ statistic. The I^2^ value of 50% or more shows significant heterogeneity. We used a random-effect model to address variability among the study findings. An I^2^ value of 25% or less indicates low heterogeneity, 25%-75% suggests moderate heterogeneity, and a value exceeding 75% indicates high heterogeneity.

Results

**Figure 1:**
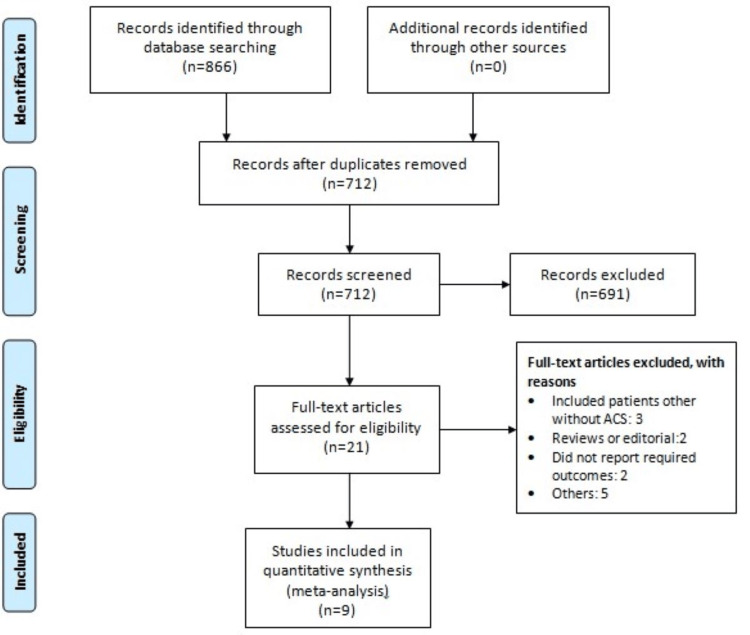
PRISMA flowchart of study selection PRISMA, Preferred Reporting Items for Systematic Reviews and Meta-Analyses; ACS, acute coronary syndrome

From the databases, a total of 866 records were retrieved. Following the elimination of duplicates (n = 154) and screening of abstracts (n = 691), 21 full-text articles were evaluated for eligibility. Among these, nine articles met the predetermined inclusion and exclusion criteria and were consequently included in the final meta-analysis. The selection process is depicted in Figure 1.

Baseline Characteristics

Among the nine studies incorporated into the quantitative synthesis, six exclusively enrolled patients diagnosed with myocardial infarction, while three also encompassed other types of acute coronary syndrome (ACS) patients. Moreover, six studies involved participants with type 2 diabetes mellitus (T2DM), whereas three studies involved individuals both with and without T2DM. The follow-up duration across the included studies varied from six to 24 months (Table 1). Table 2 presents the quality assessment of the included studies.

**Table 1:** Characteristics of the included studies SGLTi, sodium-glucose cotransporter inhibitors; NS, not specified; RCT, randomized controlled trial

Study ID	Study Design	Region	Groups	Sample Size	Follow-Up	Age (Years)	Males (n)	Diabetes (n)
Chang et al., 2022 [12]	Observational	Taiwan	SGLTi	66	23.5 Months	66.1	50	66
			Non-SGLTi	132		66.7	95	132
Chen et al., 2023 [13]	Observational	China	SGLTi	128	10 Months	64	96	128
			Non-SGLTi	104		67	79	104
Kanaoka et al., 2023 [14]	Observational	Japan	SGLTi	12955	24 Months	NS	NS	NS
			Non-SGLTi	12955				
Kurozumi et al., 2024 [15]	Observational	Japan	SGLTi	40	6 Months	65.48	32	40
			Non-SGLTi	69		73.81	50	69
Kwon et al., 2023 [16]	Observational	Korea	SGLTi	938	24 Months	56.4	769	938
			Non-SGLTi	1876		57.6	1482	1876
von Lewinski et al., 2022 [17]	RCT	Austria	SGLTi	237	12 Months	57	195	30
			Non-SGLTi	239		57	197	33
Lyu et al., 2023 [18]	Observational	Korea	SGLTi	186	12 Months	59.11	150	186
			Non-SGLTi	593		66.12	422	593
Marfella et al., 2023 [19]	Observational	Italy	SGLTi	177	12 Months	66.2	115	177
			Non-SGLTi	200		65.4	128	200
Zhu et al., 2022 [20]	Observational	China	SGLTi	141	23 Months	60.6	105	96
			Non-SGLTi	645		62.5	497	96

**Table 2:** Quality assessment of the included studies

Study ID	Selection	Comparison	Assessment	Overall
Chang et al., 2022 [12]	4	2	2	Good
Chen et al., 2023 [13]	3	2	2	Good
Kanaoka et al., 2023 [14]	3	2	3	Good
Kurozumi et al., 2024 [15]	4	2	2	Good
Kwon et al., 2023 [16]	3	1	2	Fair
Lyu et al., 2023 [18]	4	2	3	Good
Marfella et al., 2023 [19]	3	1	3	Good
Zhu et al., 2022 [20]	4	2	2	Good

Meta-Analysis of Outcomes 

**Figure 2:**
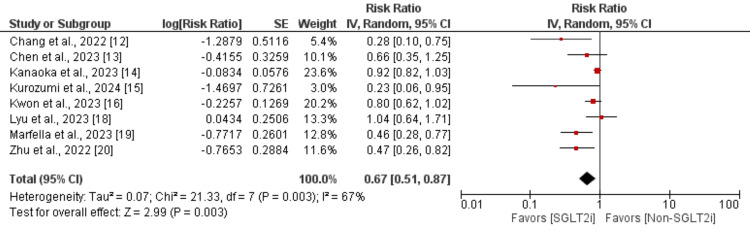
Effect of SGLT2i on MACE Sources: [12-16,18-20] SGLT2i, sodium-glucose cotransporter-2 inhibitor; MACE, major adverse cardiovascular events; IV, interval variable; CI, confidence interval; SE, standard error; df, degrees of freedom

Major adverse cardiovascular events (MACE): Eight studies assessed the risk of MACE between the sodium-glucose cotransporter inhibitor (SGLTi) and control groups in patients with ACS, and the results are shown in Figure 2. The pooled analysis of eight studies showed that the risk of MACE was significantly lower in patients receiving SGLTi compared to the control group (RR: 0.67; 95% CI: 0.51, 0.87; p-value: 0.003). Moderate heterogeneity was reported among the study results (I^2^: 67%).

**Figure 3:**
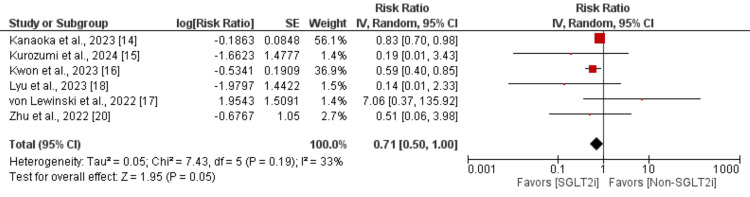
Effect of SGLT2i on all-cause mortality Sources: [14-18,20] SGLT2i, sodium-glucose cotransporter-2 inhibitors; IV, interval variable; CI, confidence interval; SE, standard error; df, degrees of freedom

All-cause mortality and cardiovascular mortality: We included six studies in the pooled analysis of comparing the risk of all-cause mortality between patients in SGLTi and the control group, and the results are shown in Figure 3. Pooled analysis showed that the risk of all-cause mortality was lower in patients receiving SGLTi compared to the control group (RR: 0.71; 95% CI: 0.50, 1.00; p-value: 0.05). Moderate heterogeneity was reported among the study results (I^2^: 33%). 

**Figure 4:**
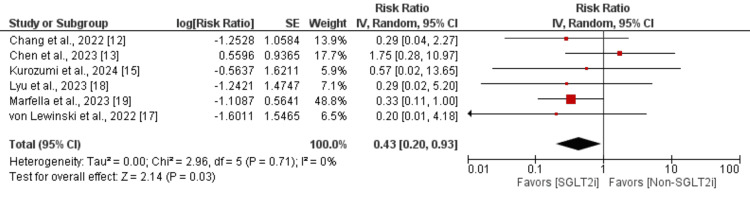
Effect of SGLT2i on cardiovascular mortality Sources: [12,13,15,17-19] SGLT2i, sodium-glucose cotransporter-2 inhibitors; IV, interval variable; CI, confidence interval; SE, standard error; df, degrees of freedom

Six studies assessed the effect of SGLTi on cardiovascular mortality in ACS patients. As shown in Figure 4, the risk of cardiovascular mortality was significantly lower in patients receiving SGLTi compared to the patients in the control group (RR: 0.43; 95% CI: 0.20, 0.93; p-value: 0.03). No significant heterogeneity was reported among the study results (I^2^: 0%).

**Figure 5:**
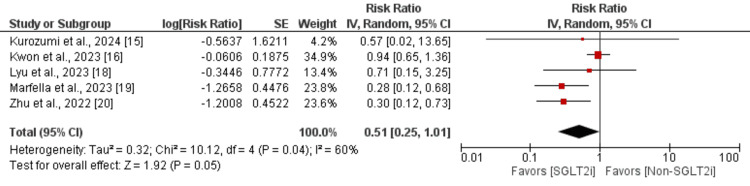
Effect of SGLT2i on recurrent ACS Sources: [15,16,18-20] SGLT2i, sodium-glucose cotransporter-2 inhibitors; IV, interval variable; CI, confidence interval; SE, standard error; df, degrees of freedom; ACS, acute coronary syndrome

**Figure 6:**
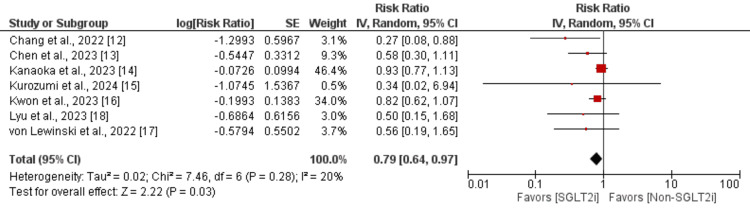
Effect of SGLT2i on hospitalization due to heart failure Sources: [12-18] SGLT2i, sodium-glucose cotransporter-2 inhibitors; IV, interval variable; CI, confidence interval; SE, standard error; df, degrees of freedom

Recurrent ACS and hospitalization for heart failure: Five studies were included in the pooled analysis to assess the impact of recurrent ACS in patients. As shown in Figure 5, the risk of developing ACS was lower in patients receiving SGLTi compared to the control group, but the difference was statistically insignificant (RR: 0.51; 95% CI: 0.25, 1.01; p-value: 0.05). Moderate heterogeneity was reported among the study results (I^2^: 60%). Moreover, as shown in Figure 6, the risk of hospitalization for cardiovascular reasons was significantly lower in patients receiving SGLTi compared to the control group (RR: 0.79; 95% CI: 0.64, 0.97; p-value: 0.03). Low heterogeneity was reported among the study results (I^2^: 20%).

Discussion

This meta-analysis aimed to evaluate the impact of SGLT2i on patients with ACS. The pooled analysis of nine studies revealed a significant reduction in the risk of MACE, all-cause mortality, cardiovascular mortality, and cardiovascular-related hospitalizations among patients receiving SGLT2i compared to those in the control group. Additionally, there was a trend toward a lower risk of recurrent ACS in the SGLT2i group, although this difference did not reach statistical significance. To the best of our knowledge, this meta-analysis represents the first comprehensive assessment of the effect of SGLT2i specifically on ACS patients. While several recently published observational studies on cardiovascular mortality have reported nonsignificant findings for all-cause mortality and cardiovascular-related hospitalization, our meta-analysis indicates a significant improvement in these outcomes when the data are combined. Our findings align with previous systematic reviews focusing on patients with T2DM [21,22], patients without T2DM [23], and reviews encompassing both patient groups [24,25].

Patients experiencing acute myocardial infarction encompass a range of risks, including the likelihood of recurrent MI, chronic heart failure (HF), life-threatening arrhythmias, and cardiovascular mortality [26,27]. The notion of promptly initiating and sustaining SGLT2 inhibition following acute MI is enticing, given the numerous proposed mechanistic effects that could potentially modify the disease's progression, susceptibility to ventricular remodeling, and development of chronic HF and advanced heart disease [28]. Recent studies conducted in experimental models of acute MI, both diabetic and nondiabetic, have demonstrated several advantages associated with SGLT2 inhibition [8]. These potential mechanisms do not primarily target coronary thrombosis but instead focus on mitigating neurohormonal activation, cardiomyocyte necrosis, and reperfusion injury [9].

The positive impact of SGLT2 inhibitors extends to certain secondary outcomes; however, our analysis revealed diverse effects compared to existing literature. Regarding all-cause mortality, we observed a significant protective effect in patients with chronic kidney disease (CKD), consistent with findings by Arnott et al. [22] yet contrasting with other reviews [29]. Furthermore, our analysis indicated a robust protective effect of SGLT2 inhibitors against hospitalization for HF, aligning with previous systematic reviews [24]. Nevertheless, most studies included in these systematic reviews and meta-analyses lacked patients with myocardial infarction, and those that did include them typically enrolled post-myocardial infarction patients, often after a 14-day period. Therefore, there is a need for additional RCTs specifically targeting myocardial infarction patients, as the effects of these drugs may differ within this population.

We were unable to conduct subgroup analysis based on the presence of T2DM due to the composition of the included studies. Of the nine studies included, six exclusively enrolled patients with T2DM, while three studies enrolled patients both with T2DM and without T2DM. However, only two out of these three studies performed subgroup analysis regarding the presence or absence of T2DM, and both studies did not report any significant difference between the two groups in terms of the efficacy of SGLT2 inhibitors. This suggests that the presence of T2DM does not alter the therapeutic effect of SGLT2 inhibitors in patients with CKD.

The Study to Evaluate the Effect of Empagliflozin on Hospitalization for Heart Failure and Mortality in Patients with Acute Myocardial Infarction (EMPACT-MI) and Dapagliflozin in Patients with MI (DAPA-MI) trials are currently ongoing clinical investigations assessing the influence of empagliflozin and dapagliflozin, respectively, on cardiovascular mortality in individuals experiencing acute myocardial infarction [21]. Our study contributes evidence indicating the effectiveness of SGLT2 inhibitors in patients with myocardial infarction. In summary, the systematic review and meta-analysis offer significant evidence regarding the potential advantages of SGLT2 inhibitors in diminishing the risk of cardiovascular events among individuals with infarction. Should further research corroborate these findings, it may prompt a substantial shift in the management of myocardial infarction patients, potentially enhancing outcomes and alleviating the disease burden.

Study Limitations

Firstly, it is important to note the absence of RCTs in this meta-analysis; all the studies included were observational. Observational studies carry a certain risk of bias. Therefore, in future investigations, more RCTs are required to validate the findings of this meta-analysis. Secondly, some baseline and outcome data were missing from the trials. As a result, we were unable to incorporate these elements into our results. For instance, we could not evaluate the impact of baseline hypoglycemic drugs in a meta-analysis because comparisons based on this variable were rarely documented in the records of the included trials. Thirdly, we could only retrieve outcome data for patients with CKD without T2DM from two trials. Therefore, we were not able to perform pooled analysis, which significantly limits the external validity of our subgroup analyses.

## Conclusions

This meta-analysis underscores the potential benefits of SGLT2 inhibitors in reducing major adverse cardiovascular events, all-cause mortality, cardiovascular mortality, and hospitalization for cardiovascular reasons in patients with acute coronary syndrome (ACS). Despite some limitations, including the absence of randomized controlled trials and limited subgroup data, our findings suggest a promising therapeutic effect of SGLT2 inhibitors in this population. Further research, particularly focusing on myocardial infarction patients, is warranted to validate these results and potentially revolutionize ACS management.

## Appendices

Table 3 shows the search strategy for PubMed.

**Table 3:** Search strategy for PubMed MeSH: Medical Subject Heading

Database	Search Strategy
PubMed	("Sodium-Glucose Transporter 2 Inhibitors" [MeSH] OR "SGLT2 Inhibitors" [MeSH]) AND ("Acute Coronary Syndrome" [MeSH] OR "Myocardial infarction" [MeSH] OR "angina" [MeSH]) AND ("Cardiovascular Diseases" [MeSH] OR "Cardiovascular Mortality" [MeSH] OR "acute coronary syndrome" [MeSH] OR "death" [MeSH] OR "all-cause mortality" [MeSH])

## References

[1] GBD 2019 Diseases and Injuries Collaborators (2020). Global burden of 369 diseases and injuries in 204 countries and territories, 1990-2019: a systematic analysis for the Global Burden of Disease Study 2019. Lancet.

[2] Hayıroğlu Mİ, Çınar T, Çiçek V, Palice A, Ayhan G, Tekkeşin Aİ (2022). The triglyceride-glucose index can predict long-term major adverse cardiovascular events in Turkish patients with high cardiovascular risk. J Lipid Atheroscler.

[3] Yahagi K, Kolodgie FD, Lutter C, Mori H, Romero ME, Finn AV, Virmani R (2017). Pathology of human coronary and carotid artery atherosclerosis and vascular calcification in diabetes mellitus. Arterioscler Thromb Vasc Biol.

[4] Ding Q, Funk M, Spatz ES, Lin H, Batten J, Wu E, Whittemore R (2022). Sex-specific impact of diabetes on all-cause mortality among adults with acute myocardial infarction: an updated systematic review and meta-analysis, 1988-2021. Front Endocrinol (Lausanne).

[5] Zinman B, Wanner C, Lachin JM (2015). Empagliflozin, cardiovascular outcomes, and mortality in type 2 diabetes. N Engl J Med.

[6] Desta L, Jernberg T, Löfman I, Hofman-Bang C, Hagerman I, Spaak J, Persson H (2015). Incidence, temporal trends, and prognostic impact of heart failure complicating acute myocardial infarction. The SWEDEHEART Registry (Swedish Web-System for Enhancement and Development of Evidence-Based Care in Heart Disease Evaluated According to Recommended Therapies): a study of 199,851 patients admitted with index acute myocardial infarctions, 1996 to 2008. JACC Heart Fail.

[7] Lee SY, Lee TW, Park GT (2021). Sodium/glucose co-transporter 2 inhibitor, empagliflozin, alleviated transient expression of SGLT2 after myocardial infarction. Korean Circ J.

[8] Liu Y, Wu M, Xu J, Xu B, Kang L (2021). Empagliflozin prevents from early cardiac injury post myocardial infarction in non-diabetic mice. Eur J Pharm Sci.

[9] Lim VG, Bell RM, Arjun S, Kolatsi-Joannou M, Long DA, Yellon DM (2019). SGLT2 inhibitor, canagliflozin, attenuates myocardial infarction in the diabetic and nondiabetic heart. JACC Basic Transl Sci.

[10] Scheen AJ (2015). Pharmacodynamics, efficacy and safety of sodium-glucose co-transporter type 2 (SGLT2) inhibitors for the treatment of type 2 diabetes mellitus. Drugs.

[11] Sayour AA, Oláh A, Ruppert M, Barta BA, Merkely B, Radovits T (2024). Effect of pharmacological selectivity of SGLT2 inhibitors on cardiovascular outcomes in patients with type 2 diabetes: a meta-analysis. Sci Rep.

[12] Chang TY, Lu CT, Huang HL (2022). Association of sodium-glucose cotransporter 2 (SGLT2) inhibitor use with cardiovascular and renal outcomes in type 2 diabetes mellitus patients with stabilized acute myocardial infarction: a propensity score matching study. Front Cardiovasc Med.

[13] Chen J, Chang J, Shi Q, Li X, Wang L, Zhao H (2023). Cardiovascular protective effect of sodium-glucose cotransporter 2 inhibitors on patients with acute coronary syndrome and type 2 diabetes mellitus: a retrospective study. BMC Cardiovasc Disord.

[14] Kanaoka K, Iwanaga Y, Nakai M (2023). Sodium-glucose cotransporter 2 inhibitor use in early-phase acute coronary syndrome with severe heart failure. Eur Heart J Cardiovasc Pharmacother.

[15] Kurozumi A, Shishido K, Yamashita T (2024). Sodium-glucose cotransporter-2 inhibitors stabilize coronary plaques in acute coronary syndrome with diabetes mellitus. Am J Cardiol.

[16] Kwon O, Myong JP, Lee Y (2023). Sodium-glucose cotransporter-2 inhibitors after acute myocardial infarction in patients with type 2 diabetes: a population-based investigation. J Am Heart Assoc.

[17] von Lewinski D, Kolesnik E, Tripolt NJ (2022). Empagliflozin in acute myocardial infarction: the EMMY trial. Eur Heart J.

[18] Lyu YS, Oh S, Kim JH, Kim SY, Jeong MH (2023). Comparison of SGLT2 inhibitors with DPP-4 inhibitors combined with metformin in patients with acute myocardial infarction and diabetes mellitus. Cardiovasc Diabetol.

[19] Marfella R, Sardu C, D'Onofrio N (2023). SGLT-2 inhibitors and in-stent restenosis-related events after acute myocardial infarction: an observational study in patients with type 2 diabetes. BMC Med.

[20] Zhu Y, Zhang JL, Yan XJ, Sun L, Ji Y, Wang FF (2022). Effect of dapagliflozin on the prognosis of patients with acute myocardial infarction undergoing percutaneous coronary intervention. Cardiovasc Diabetol.

[21] Zelniker TA, Wiviott SD, Raz I (2019). SGLT2 inhibitors for primary and secondary prevention of cardiovascular and renal outcomes in type 2 diabetes: a systematic review and meta-analysis of cardiovascular outcome trials. Lancet.

[22] Arnott C, Li Q, Kang A (2020). Sodium-glucose cotransporter 2 inhibition for the prevention of cardiovascular events in patients with type 2 diabetes mellitus: a systematic review and meta-analysis. J Am Heart Assoc.

[23] Tsai WC, Hsu SP, Chiu YL (2022). Cardiovascular and renal efficacy and safety of sodium-glucose cotransporter-2 inhibitors in patients without diabetes: a systematic review and meta-analysis of randomised placebo-controlled trials. BMJ Open.

[24] Salah HM, Al'Aref SJ, Khan MS (2021). Effect of sodium-glucose cotransporter 2 inhibitors on cardiovascular and kidney outcomes-systematic review and meta-analysis of randomized placebo-controlled trials. Am Heart J.

[25] Chen HB, Yang YL, Yu TH, Li YH (2022). SGLT2 inhibitors for the composite of cardiorenal outcome in patients with chronic kidney disease: a systematic review and meta-analysis of randomized controlled trials. Eur J Pharmacol.

[26] Wellings J, Kostis JB, Sargsyan D, Cabrera J, Kostis WJ (2018). Risk factors and trends in incidence of heart failure following acute myocardial infarction. Am J Cardiol.

[27] Peters SA, Colantonio LD, Dai Y (2021). Trends in recurrent coronary heart disease after myocardial infarction among us women and men between 2008 and 2017. Circulation.

[28] Verma S, Anker SD, Butler J, Bhatt DL (2020). Early initiation of SGLT2 inhibitors is important, irrespective of ejection fraction: SOLOIST‐WHF in perspective. ESC Heart Fail.

[29] Li N, Zhou G, Zheng Y (2022). Effects of SGLT2 inhibitors on cardiovascular outcomes in patients with stage 3/4 CKD: a meta-analysis. PLoS One.

